# Phagocytic astrocytes: Emerging from the shadows of microglia

**DOI:** 10.1002/glia.24145

**Published:** 2022-02-10

**Authors:** Hiroyuki Konishi, Schuichi Koizumi, Hiroshi Kiyama

**Affiliations:** ^1^ Department of Functional Anatomy and Neuroscience Nagoya University Graduate School of Medicine Nagoya Japan; ^2^ Department of Neuropharmacology University of Yamanashi Yamanashi Japan; ^3^ GLIA Center, Interdisciplinary Graduate School of Medicine University of Yamanashi Yamanashi Japan

**Keywords:** astrocyte, clearance, microglia, phagocyte, phagocytic receptor, phagocytosis, synapse

## Abstract

Elimination of dead or live cells take place in both a healthy and diseased central nervous system (CNS). Dying or dead cells are quickly cleared by phagocytosis for the maintenance of a healthy CNS or for recovery after injury. Live cells or parts thereof, such as the synapses and myelin, are appropriately eliminated by phagocytosis to maintain or refine neural networks during development and adulthood. Microglia, the specific population of resident macrophages in the CNS, are classically considered as primary phagocytes; however, astrocytes have also been highlighted as phagocytes in the last decade. Phagocytic targets and receptors are reported to be mostly common between astrocytes and microglia, which raises the question of how astrocytic phagocytosis differs from microglial phagocytosis, and how these two phagocytic systems cooperate. In this review, we address the consequences of astrocytic phagocytosis, particularly focusing on these elusive points.

## Main Points

- Phagocytic targets are mostly common between astrocytes and microglia.
- Various factors determine whether either of the glial cell types phagocytose the targets.
- Interaction of astrocytes and microglia may provide microglia with a phagocytic advantage.

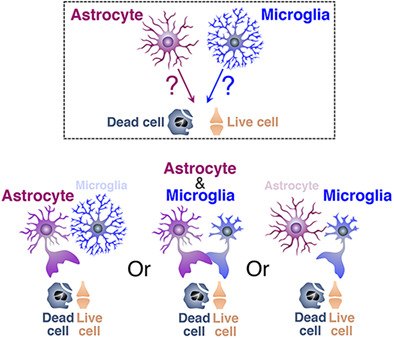

## 1 INTRODUCTION

Many cells die in the central nervous system (CNS) during development and disease (Bredesen et al., 2006; Oppenheim, 1991). Cell death also occurs in a healthy adult CNS at low frequencies, which increases with age (Pakkenberg & Gundersen, 1997; West, 1993). Apoptosis is a process of programmed cell death, whereby cells are quickly removed by phagocytes with retention of the plasma membrane integrity (Kerr et al., 1972). However, if apoptotic cells fail to be cleared, they transform into necrotic cells, with a breakdown of the plasma membrane and leakage of the intracellular molecules (e.g., damage‐associated molecular patterns [DAMPs]), which triggers an inflammatory response in the surrounding glial cells and eventually leads to tissue inflammation and damage (Nagata, 2018; Sierra et al., 2013). In addition to inducing an inflammatory *milieu*, the accumulated cell debris, such as those of axons and myelin, can be a physical or molecular barrier to the growth of axons during development and disease (Chen et al., 2000; Schwab & Caroni, 1988; Tanaka et al., 2009). Therefore, appropriate clearance of dead cells by phagocytosis is necessary for the development, maintenance, and regeneration of the CNS. In addition to dying or dead cells, whole or parts of live cells are eliminated by phagocytosis. For instance, synaptic connections are initially overbuilt during development, and phagocytic pruning of unnecessary synapses is essential for the establishment of proper neural networks (Chung et al., 2013; Paolicelli et al., 2011; Rakic et al., 1986; Riccomagno & Kolodkin, 2015). Synaptic formation and elimination also occur in adulthood in an experience‐dependent manner (Trachtenberg et al., 2002).

Microglia, the resident mononuclear cells of the CNS (Ajami et al., 2007; Ginhoux et al., 2010; Mildner et al., 2007), are regarded as the main phagocytes in the CNS because of their high phagocytic capacity (del Rio‐Hortega, 1932; Marquez‐Ropero et al., 2020; Wolf et al., 2017). However, the phagocytic capacity of astrocytes, the multifunctional glial cells in the CNS (Abbott et al., 2006; Araque et al., 1999; Attwell et al., 2010; Sofroniew & Vinters, 2010; Verkhratsky & Nedergaard, 2018), has been highlighted in the last decade. A breakthrough might be the study showing that astrocytes eliminate synapses by phagocytosis in the developing brain (Chung et al., 2013). Following this study, the phagocytic activity of astrocytes has been in the spotlight, with reports of astrocytic phagocytosis increasing over the last few years. Accordingly, astrocytes, in addition to microglia, are established as CNS phagocytes that clear dead cells and parts of live cells, such as synapses and axons (Jung & Chung, 2018). However, most of these phagocytic targets of astrocytes can also be phagocytosed by microglia, raising the question of how astrocytic phagocytosis differs from microglial phagocytosis, and how these two phagocytic systems cooperate. Recently, many review articles have described the phagocytic activity of astrocytes; however, the difference or coordination between astrocytes and microglia has been discussed less. Therefore, in this review, we address astrocytic phagocytosis by highlighting these elusive points. Astrocytes also take up extracellular protein aggregates, such as β‐amyloid (Aβ) and α‐synuclein (Koistinaho et al., 2004; H. J. Lee et al., 2008; Wakabayashi et al., 2000; Yamaguchi et al., 1998). In this review, however, we confined our scope to phagocytosis of live or dead cells because of the prominent advances in recent studies.

## 2 PHAGOCYTIC TARGETS AND THEIR RECOGNITION RECEPTORS EXPRESSED BY ASTROCYTES AND MICROGLIA

**FIGURE 1:**
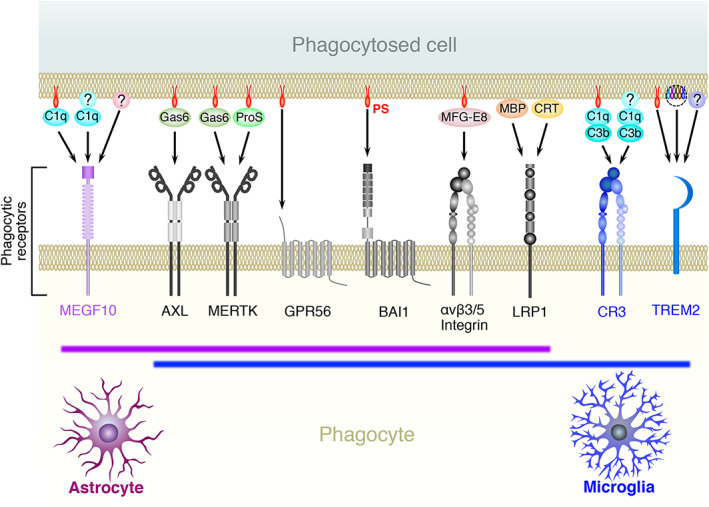
Major phagocytic receptors for the recognition of live or dead cells by astrocytes or microglia. Astrocyte‐ or microglia‐specific receptors are shown in magenta and blue, respectively. Receptors commonly expressed by both glial cells are shown in black and white. The ligand(s) for each phagocytic receptor are also described

The major phagocytic receptors and adaptor molecules, with which astrocytes and microglia recognize phagocytic targets, are shown in Figure 1. The phagocytic targets, their recognition receptors, and key references are shown in Table 1 (dead cells) and Table 2 (live cells). Most studies presented in Tables 1 and 2 were performed using rodents; however, all the phagocytic receptors described also exist in humans, suggesting similar operation of these phagocytic systems in humans.

**TABLE 1:** Phagocytic targets (dead cells) and responsible phagocytic receptors

**Astrocytes**		**Microglia**	
**Dead cell**		** Dead cell**	
AXL	Konishi et al., 2020	AXL	Fourgeaud et al., 2016 Tufail et al., 2017 Ayata et al., 2018
MERTK	Konishi et al., 2020	MERTK	Fourgeaud et al., 2016 Tufail et al., 2017 Ayata et al., 2018 Diaz‐Aparicio et al., 2020
BAI1	Park et al., 2007	BAI1	Mazaheri et al., 2014
MEGF10	Tasdemir‐Yilmaz & Freeman, 2013 Iram et al., 2016 Morizawa et al., 2017	αvβ3/5 integrin	Liu et al., 2013 Fuller et al., 2018
		TREM2	Takahashi et al., 2005
		CR3	Silverman et al., 2019
			
**Dystrophic or degenerated myelin**		** Dystrophic or degenerated myelin**	
LRP1	Gaultier et al., 2009 Ponath et al., 2017	LRP1	Gaultier et al., 2009
αvβ3/5 integrin	Mills et al., 2015	AXL	Hoehn et al., 2008
		MERTK	Shen et al., 2021
		TREM2	Cantoni et al., 2015 Cignarella et al., 2020
		CR3	Bruck & Friede, 1990 Liu et al., 2020

**TABLE 2:** Phagocytic targets (live cells) and responsible phagocytic receptors

**Astrocytes**		**Microglia**	
		**Live neuron**	
		MERTK	Neher et al., 2013
		LRP1	Fricker et al., 2012
		αvβ3/5 integrin	Fricker et al., 2012 Neniskyte & Brown, 2012 Neher et al., 2013
		CR3	Anderson et al., 2019
			
**Live synapse**		**Live synapse**	
MERTK	Chung et al., 2013	MERTK	Chung et al., 2013 Park et al., 2021
MEGF10	Chung et al., 2013 Lee et al., 2021	TREM2	Filipello et al., 2018 Scott‐Hewitt et al., 2020
		CR3	Schafer et al., 2012 Hong et al., 2016
		GPR56	Li et al., 2020 Li et al., 2021
			
**Live axon**		**Live axon**	
?	Nguyen et al., 2011 Davis et al., 2014	CR3	Lim & Ruthazer, 2021
		?	Maeda et al., 2010
			
		**Live neuronal or glial progenitor/precursor cell**	
		CR3	VanRyzin et al., 2019
		?	Cunningham et al., 2013 Nelson et al., 2017
			
**Live astrocyte**		**Live astrocyte**	
?	Punal et al., 2019	?	Punal et al., 2019
			
		**Live astrocytic endfeet**	
		?	Haruwaka et al., 2019
			
		**Live myelin**	
		?	Maeda et al., 2010 Hughes & Appel, 2020
			
		**Live oligodendrocyte progenitor cell**	
		?	Nemes‐Baran et al., 2020
			
		**Live photoreceptor**	
		αvβ3/5 integrin	Zhao et al., 2015
			
		**Live endothelial cell**	
		?	Jolivel et al., 2015
			
		**Live tumor cell**	
		?	Hutter et al., 2019

Astrocytic phagocytosis was reported in the mammalian brain more than half a century ago (Colonnier, 1964; McMahan, 1967; Mugnaini & Walberg, 1967); however, the molecules involved were mostly unknown until Cahoy et al. (Cahoy et al., 2008) performed microarray analysis of isolated astrocytes. This pioneering study identified several phagocytic receptors, including AXL and MERTK, both of which are members of the TYRO3/AXL/MERTK (TAM) family phagocytic receptors (Lemke, 2013), MEGF10, which is a mammalian homolog of *Drosophila* phagocytic receptor Draper (Freeman et al., 2003), αvβ5 integrin (Finnemann et al., 1997), and LRP1 (Gardai et al., 2005). Furthermore, the study also found several cytosolic or transmembrane molecules, which are homologous to *C. elegans* cell death‐related genes regulating phagocytic activity, including Crk1 (ced‐2), Dock1 (ced‐5), Gulp1 (ced‐6), ABCA1 (ced‐7), Rac1 (ced‐10), and Elmo2 (ced‐12) (Cahoy et al., 2008), evaluating astrocytes as phagocytes in the context of gene expression profiles.

### 2.1 Phagocytic receptors for dead cells or degenerated cellular parts

#### 2.1.1 Dead cell receptors

Iram et al. (Iram, Ramirez‐Ortiz, et al., 2016) demonstrated that the astrocyte‐specific molecule MEGF10 is a phagocytic receptor for apoptotic cells, which was supported by their finding that the number of apoptotic cells was increased in *Megf10*‐deficient neonatal brains. The universal feature of apoptotic cells is the exposure of phosphatidylserine (PS) on the cell surface (Fadok et al., 1992; Nagata, 2018). MEGF10 is thought not to bind PS directly; instead, it recognizes PS using the complement protein C1q as an adaptor (Iram, Ramirez‐Ortiz, et al., 2016). However, because a study denied involvement of C1q when astrocytic MEGF10 recognizes unnecessary synapses, as described in a later section (Chung et al., 2013), additional adaptor molecule(s) bridging PS and MEGF10 might exist. Our study also found that astrocytic MEGF10 is a phagocytic receptor for dead cells that accumulate massively after ischemic brain injury, although it is unclear whether the engulfed cells die by apoptosis or not (Morizawa et al., 2017). In line with these reports, Draper, the *Drosophila* homolog of MEGF10 (Freeman et al., 2003), participates in the clearance of axonal debris during metamorphosis in *Drosophila* (Tasdemir‐Yilmaz & Freeman, 2014). In addition to MEGF10, TAM family receptor tyrosine kinase (Lemke, 2013), which recognizes PS via bridging molecules Gas6 or ProS, plays a role in astrocytic phagocytosis of apoptotic cells. Among TAM family members, our study demonstrated that AXL and MERTK are essential molecules for phagocytic removal of apoptotic cells by astrocytes (Konishi et al., 2020). Moreover, BAI1, a member of the adhesion family of GPCRs, also functions as a phagocytic receptor in astrocytes by directly binding to PS exposed on the apoptotic cell surface (D. Park et al., 2007).

As is the case with astrocytes, microglia use AXL, MERTK, and BAI1 to recognize apoptotic cells (Ayata et al., 2018; Diaz‐Aparicio et al., 2020; Fourgeaud et al., 2016; Mazaheri et al., 2014, Tufail et al., 2017). In addition, αvβ3 or αvβ5 integrin, both of which are known as vitronectin receptors (Wayner et al., 1991), also function as a microglial phagocytic receptor for apoptotic cells by indirect recognition of PS via the PS‐binding molecule MFG‐E8 (Fuller & Van Eldik, 2008; Liu et al., 2013). Besides these receptors, which are equipped with both glial cells, microglia possess unique phagocytic receptors for dead cells. Microglia, not astrocytes, express TREM2, whose mutation is highly associated with dementia through microglial hypoactivation in humans (Guerreiro et al., 2013; Jonsson et al., 2013; Paloneva et al., 2002). Microglial TREM2 is shown to be a phagocytic receptor for apoptotic cells (Takahashi et al., 2005), and dementia‐associated mutations of TREM2 such as T66M impair phagocytic clearance of apoptotic cells in human induced pluripotent stem cell‐derived microglia (Garcia‐Reitboeck et al., 2018). Given that TREM2 can bind various lipid species, including PS (Y. Wang et al., 2015), TREM2 may directly bind to PS exposed on the apoptotic cell surface. Microglia also specifically express complement molecule C3 receptor (CR3) (Schafer et al., 2012), the complex of CD11b and CD18 (Michishita et al., 1993). In addition to acting as a key phagocytic receptor for opsonized synapses as described in a later section, CR3 also participates in the clearance of apoptotic cells. A mouse model of photoreceptor degeneration showed that microglial CR3 recognizes apoptotic photoreceptors opsonized with the CR3 ligand, iC3b (Silverman et al., 2019). Regarding the regulation of microglial phagocytosis of dead cells, a study investigating some brain regions with high rates of spontaneous neuronal death in adult mice showed an intriguing possibility that microglia upregulate phagocytosis‐related genes via an epigenetic program when they encounter dying cells (Ayata et al., 2018). In line with this report, microglia upregulate phagocytosis‐related genes to actively phagocytose dead cells in specific regions of the developing CNS, where developmental cell death frequently occurs, such as within the white matter of early postnatal rodents (Hammond et al., 2019; Q. Li et al., 2019; Trapp et al., 1997). Microglia with phagocytosis‐related genes were also found in the white matter of aged mice, where damaged myelin accumulated (Safaiyan et al., 2021). Therefore, brain regions, in association with their age‐related changes, significantly affect microglial phagocytic activity.

#### 2.1.2 Dystrophic or degenerated myelin receptors

Myelin sheath, the functional compartment of oligodendrocytes, undergoes degradation in demyelinating diseases or after CNS injury (Stadelmann et al., 2019). Phagocytes undertake clearance of myelin debris, whose impairment accelerates disease pathology (Cantoni et al., 2015; Lampron et al., 2015; Shen et al., 2021). Astrocytes actively phagocytose myelin debris in various autopsy samples during demyelination, as well as in a rat model of spinal cord injury (Ponath et al., 2017; S. Wang et al., 2020). Culture experiments suggested that astrocytes possibly take up myelin debris by LRP1 (Ponath et al., 2017), which can directly bind various myelin‐specific proteins, including MBP (Fernandez‐Castaneda et al., 2013; Gaultier et al., 2009). In addition to these pathological conditions, astrocytes also phagocytose dystrophic myelin of the optic nerve during the metamorphic remodeling of *Xenopus laevis* by recognition of myelin surface‐exposed PS using the MFG‐E8‐integrin system (Mills et al., 2015).

Microglia also likely use LRP1 to engulf myelin debris (Gaultier et al., 2009). In addition, MERTK, a member of the TAM family, is a microglial phagocytic receptor for degenerated myelin (Healy et al., 2016; Shen et al., 2021), presumably by the recognition of PS exposed on myelin debris via Gas6 or ProS (Glade & Smith, 2015). Impairment of another TAM family member, AXL, results in the accumulation of myelin debris in an animal model of demyelination (Hoehn et al., 2008), suggesting that AXL may play a synergistic role with MERTK. In addition to LRP1, AXL, and MERTK, all of which are also equipped with astrocytes (Cahoy et al., 2008; Konishi et al., 2020), microglia‐specific phagocytic receptors, TREM2 and CR3, both of which can also recognize apoptotic cells as described in a previous section, play significant roles in myelin clearance. TREM2 may stimulate the uptake of myelin debris by directly binding to myelin lipids (Cantoni et al., 2015; Cignarella et al., 2020), given that TREM2 can recognize various lipid species, including sulfatides and sphingomyelin, which are thought to be exposed on damaged myelin (Poliani et al., 2015; Y. Wang et al., 2015). Microglial CR3 recognizes myelin debris opsonized with C3b/iC3b to induce myelin phagocytosis (Bruck & Friede, 1990; Liu et al., 2020; Vanguri et al., 1982).

### 2.2 Phagocytic receptors for live cells or their parts

#### 2.2.1 Synapse receptors

During development, excess synapses are initially formed (Rakic et al., 1986), and weak synapses are eliminated by synaptic pruning to refine neural networks (Riccomagno & Kolodkin, 2015). In adulthood, synapse elimination and formation constantly occur during the modulation of neural circuits (Trachtenberg et al., 2002). Nearly a decade ago, both astrocytes and microglia emerged as pivotal players in synaptic pruning (Chung et al., 2013; Paolicelli et al., 2011; Tremblay et al., 2010). Chung et al. (Chung et al., 2013) revealed that astrocytic MEGF10 and MERTK, both of which are astrocytic phagocytic receptors for dead cells, cooperate when astrocytes eliminate synapses in the lateral geniculate nucleus during postnatal development. Very recently, the same group reported the significance of MEGF10 in synaptic pruning in adult mice. Astrocytes eliminate excitatory synapses through MEGF10 in the adult hippocampus to maintain circuit homeostasis or form memories (J. H. Lee et al., 2021). Although the mechanisms underlying the synaptic tagging are under debate, recent studies suggest that the trigger is PS exposure on the synaptic surface, similar to the *eat‐me signal* on the apoptotic cell surface (Gyorffy et al., 2018; T. Li et al., 2020; T. Li et al., 2021; J. Park et al., 2021; Scott‐Hewitt et al., 2020). Therefore, it is likely that MERTK recognizes PS on the synaptic surface via Gas6 or ProS (Lemke, 2013). Although a study on astrocytic phagocytosis of apoptotic cells found that astrocytic MEGF10 recognized PS exposed on apoptotic cells via C1q (Iram, Ramirez‐Ortiz, et al., 2016), Chung et al. (Chung et al., 2013) denied the involvement of C1q in MEGF10‐dependent synaptic phagocytosis. Therefore, synaptic tag(s) other than PS or other bridging molecule(s) between MEGF10 and PS may exist.

In contrast to astrocytes, microglia do not express MEGF10 (Chung et al., 2013). However, they highly express MERTK, using it as a phagocytic receptor for synapses similar to astrocytes (Chung et al., 2013; J. Park et al., 2021). In addition to classically known phagocytic receptors, recent studies identified an isoform of GPR56, which belongs to an adhesion family of GPCRs, as a novel PS receptor (T. Li et al., 2020; T. Li et al., 2021). Direct binding of microglial GPR56 to the synaptic surface PS triggers synaptic elimination during development. As GPR56 is also highly expressed by astrocytes (Chiou et al., 2021), astrocytic GPR56 may play the same role. In addition to MERTK and GPR56, both of which are expressed in both astrocytes and microglia, microglia use microglia‐specific phagocytic receptors, TREM2 and CR3, for synapse elimination, as is the case with phagocytosis of dead cells. Filipello et al. (Filipello et al., 2018) found that *Trem2*‐deficient microglia showed reduced phagocytic activity during synaptic elimination in the developmental hippocampus. TREM2 can bind to various lipid species, including PS (Y. Wang et al., 2015), suggesting that TREM2 directly binds to the synaptic surface PS exposed on the surface of unnecessary synapses to engulf them (Scott‐Hewitt et al., 2020). Regarding complement‐mediated synaptic elimination, the binding of C1q to PS exposed on unnecessary synapses is expected as the initial trigger that stimulates local accumulation of C3b/iC3b (Linnartz et al., 2012; Scott‐Hewitt et al., 2020). Microglia then recognize deposited C3b via CR3 to perform synaptic phagocytosis (Hong et al., 2016; Schafer et al., 2012).

As discussed, both astrocytes and microglia phagocytose synapses, and some responsible phagocytic receptors have been identified. However, it remains under debate how glial cells contact and take up synapses; in other words, whether glial cells eliminate synapses solely by phagocytosis. Phagocytosis is defined as engulfment of particles larger than 500 nm (Flannagan et al., 2012). A study demonstrated that microglia nibbled off small fragments of presynaptic elements with an average size of 250 nm in postnatal hippocampal development (Weinhard et al., 2018). This process is like trogocytosis, also known as partial phagocytosis, which has been studied in the immune system (Joly & Hudrisier, 2003). Synaptic trogocytosis by microglia does not require microglial phagocytic receptor CR3 in mice (Weinhard et al., 2018), in contrast to developmental synaptic phagocytosis (Schafer et al., 2012), suggesting that molecular mechanisms of microglial synaptic engulfment may differ between phagocytosis and trogocytosis. Further studies, including those exploring whether astrocytes also perform trogocytosis, are needed to reveal the mechanisms of synaptic engulfment by glial cells.

#### 2.2.2 Receptors for whole live cells or their parts (except synapses)

Astrocytes have been reported to engulf other parts of live cells apart from neuronal synapses. Even in a healthy condition, the optic nerve head of mice contains astrocytes that unexpectedly express the phagocytosis‐related molecule MAC‐2/galectin‐3 (Nguyen et al., 2011), whose expression is normally observed in microglia but not in astrocytes (Komine et al., 2018; Morizawa et al., 2017). As expected by the expression of MAC‐2, astrocytes in the optic nerve head constantly phagocytose the protrusion of live optic nerves, which are sometimes formed within the axons and contain axonal components such as mitochondria and microtubules (Davis et al., 2014; Nguyen et al., 2011). This specific type of astrocyte expresses the phagocytic receptor LRP1 and PS‐binding molecule MFG‐E8 (Nguyen et al., 2011), supporting the appearance of their phagocytic capacity. Besides the live axonal compartment, a study in the developing retina reported that astrocytes engulfed live neighboring astrocytes themselves, which did not show cell death features, although phagocytic receptors involved were not revealed (Punal et al., 2019).

Microglia can also phagocytose other parts of live cells apart from neuronal synapses. In a mouse model of ischemic brain injury, microglia became associated with blood vessels and phagocytosed a part of live endothelial cells in the peri‐infarct region, called the penumbra, promoting vessel disintegration (Jolivel et al., 2015). In the later stage of sustained inflammation in mice, activated microglia phagocytosed astrocytic endfeet, probably in a viable state, reducing blood–brain barrier integrity (Haruwaka et al., 2019). Therefore, microglial phagocytosis of live blood–brain barrier components contributes to its breakdown in some pathological conditions. In a nerve injury‐induced neuropathic pain model, our previous study reported that activated microglia engulfed live myelinated axons in the spinal dorsal horn, possibly contributing to the pathogenesis of neuropathic pain, although phagocytic receptors were not addressed in this study (Maeda et al., 2010). A recent study demonstrated that microglial trogocytosis pruned live axon terminals to suppress axonal arborization in developing *Xenopus laevis*. This developmental “axonal pruning” in *Xenopus laevis* is likely mediated by complement tagging of axons and its recognition by microglial receptors for C3, similar to synaptic pruning (Lim & Ruthazer, 2021). During development, “myelin pruning” is also undertaken by microglia. Microglia engulf the myelin sheath but not the oligodendrocyte cell body in a neuronal activity‐dependent manner to refine the myelin sheath in zebrafish (Hughes & Appel, 2020). Besides these parts of live cells, microglia can engulf whole live cells such as neurons, glial cells, and their progenitor/precursor cells (Alawieh et al., 2018; Anderson et al., 2019; Cunningham et al., 2013; Fricker, Neher, et al., 2012; Luo et al., 2016; Neher et al., 2013; Nelson et al., 2017; Nemes‐Baran et al., 2020; Neniskyte & Brown, 2013; Punal et al., 2019; VanRyzin et al., 2019). Studies suggest that stressed viable neurons expose PS on their surface (Neher et al., 2011), which is recognized by microglial MERTK and αvβ3/5 integrin via PS‐binding molecules Gas6/ProS and MFG‐E8, respectively (Fricker, Neher, et al., 2012; Neher et al., 2013; Neniskyte & Brown, 2013). Microglial CR3 is also involved in phagocytic clearance of live neurons, which was revealed by a study of embryonic development of retinal ganglion cell (Anderson et al., 2019). Microglial recognition of neuronal surface calreticulin, which is normally an endoplasmic reticulum protein that is exposed on the cell surface in inflammatory conditions, by LRP1 may play an additional role (Fricker, Oliva‐Martin, & Brown, 2012; Gardai et al., 2005). In addition to neurons, microglia engulf live neural precursor cells in the ventricular zone/subventricular zone of the developing brain to reduce the size of the neural precursor cell pool (Cunningham et al., 2013). A study also showed that microglia phagocytose live neural progenitor cells in the neonatal hippocampus (Nelson et al., 2017). In the study, male mice had less phagocytic microglia than female mice, which was regulated by sex hormone, suggesting the possibility that sexual differences in microglial phagocytic activity may contribute to lifelong sexual differences in hippocampal function. Although the consequence of sexual differences on microglial phagocytic activity is different, a study reported that androgen promoted microglial phagocytosis of viable newborn cells, which largely differentiated into astrocytes, in the neonatal amygdala in a complement‐dependent manner (VanRyzin et al., 2019). As a result, male rats had fewer astrocytes in the amygdala, contributing to male social play. Therefore, sex hormone‐regulated microglial phagocytosis of live progenitor or newborn cells during development may affect not only cellular composition, but also functions in some brain regions. Besides whole neurons, glial cells, and their progenitor/precursor cells, soma of stressed photoreceptors are engulfed alive by microglia in a mouse model of retinitis pigmentosa, accelerating retinal degeneration (L. Zhao et al., 2015). Microglia can also phagocytose tumor cells (Hutter et al., 2019). Tumor cells express *don't eat me signal* CD47 in a glioblastoma multiforme model, to prevent being cleared by microglial phagocytosis. However, live tumor cells are actively phagocytosed by microglia after administration of functional blocking antibody for CD47.

## 3 FACTORS WHICH DETERMINE EITHER ASTROCYTIC OR MICROGLIAL PHAGOCYTOSIS

### 3.1 Factors in the phagocytosis of dead cells

**FIGURE 2:**
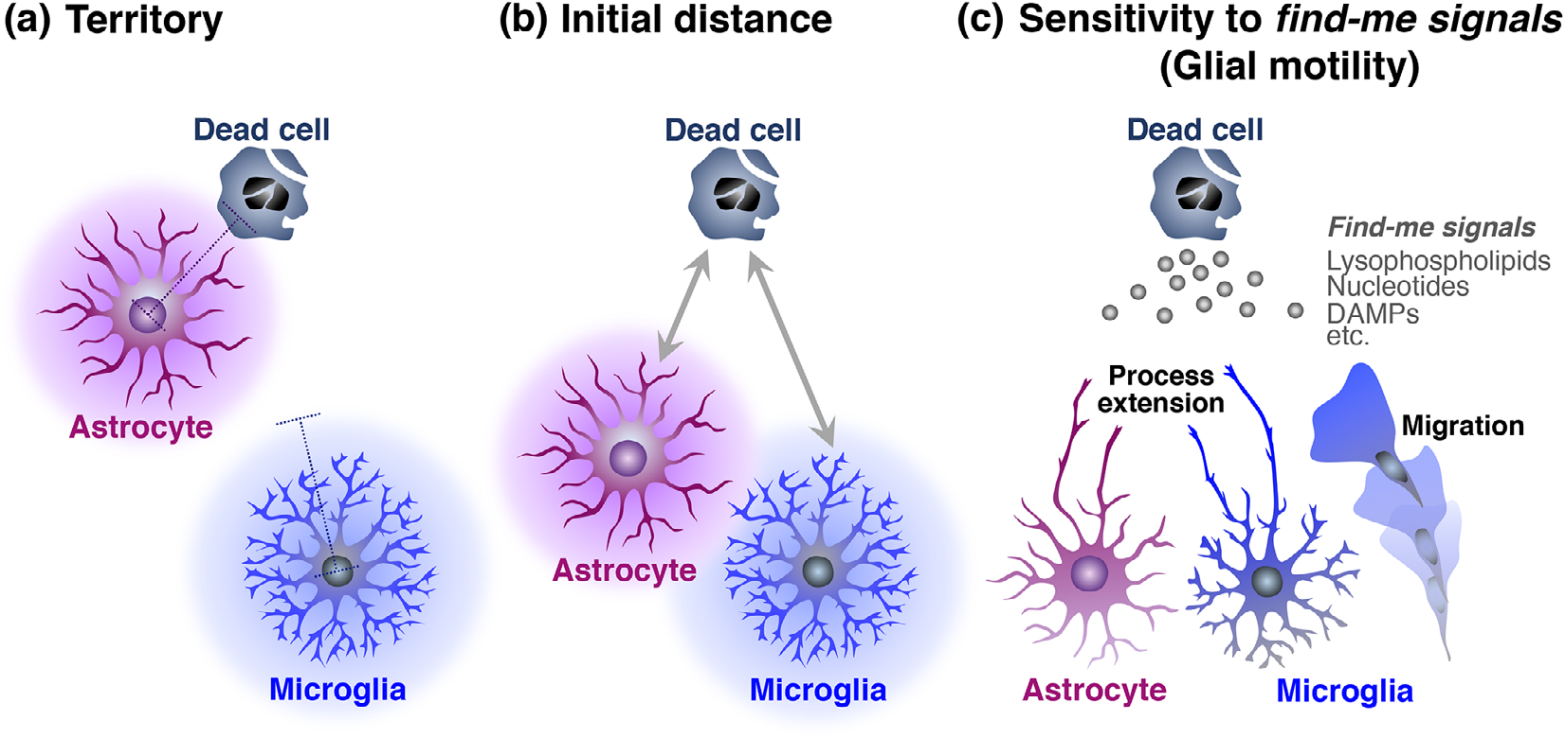
Factors which determine either astrocytic or microglial phagocytosis of dead cells. (a) Phagocytic territory of each glia. (b) Initial distance between each glia and dead cells. (c) Sensitivity of each glia to *find‐me signals*, such as lysophospholipids, nucleotides, and DAMPs, released from dead cells, which determines glial motility

Both astrocytes and microglia possess multiple phagocytic receptors that recognize dead cells (Figure 1), raising the question of what determines whether dead cells are engulfed by astrocytes or microglia. Studies using cultured astrocytes indicated that speed of both uptake and digestion of cell debris was significantly low in astrocytes (Loov et al., 2015; Magnus et al., 2002). In addition, astrocytes engulf a smaller size of cell debris than microglia in vivo (Damisah et al., 2020; Morizawa et al., 2017). Thus, the astrocytic capacity for the clearance of dead cells is expected to be lower than that of microglia. However, in vivo studies have reported situations in which astrocytes, rather than microglia, predominantly engulfed dying or dead cells (Damisah et al., 2020; Morizawa et al., 2017). Our study revealed that after brain ischemia, reactive astrocytes were mainly found in the penumbra, where astrocytes actively phagocytosed dead cells (Morizawa et al., 2017). In contrast, microglia accumulated significantly and phagocytosed in the ischemic core region, indicating a different territory between astrocytes and microglia (Figure 2a). This territorial difference between astrocytes and microglia has been shown microenvironmentally by a recent study using intravital live imaging of astrocytes and microglia around apoptotic neurons (Figure 2a) (Damisah et al., 2020). Upon induction of apoptosis to single neurons, neuronal cell bodies were engulfed by microglia, whereas neurites were engulfed by astrocytes. Given that astrocytes have extensively arborized processes, which are frequently close to neurites in physiological conditions, the authors suggested that the closeness between glial cells and their phagocytic target may be a critical determinant. This study further demonstrated that astrocytes phagocytosed the cell body of apoptotic neurons instead of microglia when microglia were pharmacologically depleted (Damisah et al., 2020). Other studies, including ours, have also shown similar compensatory phagocytic action of astrocytes in the absence or dysfunction of microglia (Abiega et al., 2016; Konishi et al., 2020; Punal et al., 2019). Taken together, these results and the notion that PS exposure on the surface of dead cells is the main cue for phagocytic clearance, regardless of the mode of cell death (i.e., apoptosis or necrosis) (D'Arcy, 2019; Fadok et al., 1992; Nagata, 2018; Westman et al., 2019), dead cells may not have a strict selectivity whether they are engulfed by astrocytes or microglia if the dead cells are close to both types of glial cells. Glial cells, which have the advantage of reaching and surrounding dead cells, are likely to phagocytose them (Damisah et al., 2020). In addition to the initial distance between glial cells and dead cells (Figure 2b), sensitivity to the *find‐me signals* leaked from dying or dead cells, such as lysophospholipids (e.g., lysophosphatidylcholine and S1P) (Gude et al., 2008; Lauber et al., 2003), nucleotides (e.g., DNA, RNA, and ATP/UTP/UDP) (Elliott et al., 2009; Koizumi et al., 2007), and intracellular proteins (DAMPs including HMGB1 and heat shock proteins) (Venereau et al., 2015), could be critical factors because they determine the speed of migration or process extension of glial cells (Figure 2c).

### 3.2 Factors in synaptic phagocytosis

Although both astrocytes and microglia can phagocytose synapses, several studies have reported situations where synapses are preferentially phagocytosed by astrocytes. In the hippocampal CA1 region of normal adult mice, the number of internalized synapses is larger within astrocytes than microglia, indicating that astrocytic phagocytosis is predominant (J. H. Lee et al., 2021). In a mouse model of Alzheimer's disease (AD) (APP/PS1 mice), presynaptic dystrophies were completely engulfed by amyloid plaque‐associated astrocytes, whereas microglia were also recruited to the plaque but did not engulf presynaptic dystrophies (Gomez‐Arboledas et al., 2018). In a mouse model of acute sleep deprivation, astrocytic phagocytosis of synapses, but not microglial phagocytosis, was promoted in the cerebral cortex (Bellesi et al., 2017).

**FIGURE 3:**
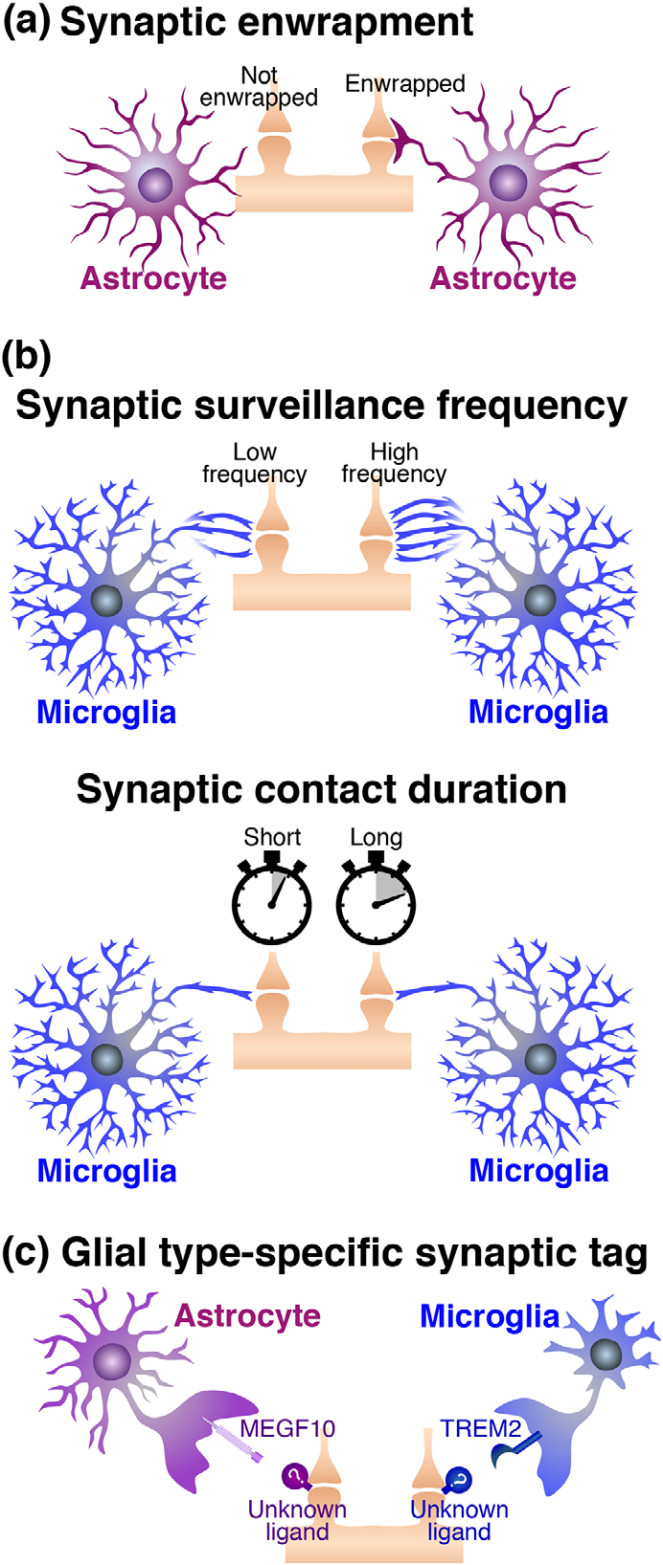
Factors which are important for determination of either astrocytic or microglial phagocytosis of synapses. (a) Synaptic enwrapment by astrocytes. (b) Synaptic surveillance frequency by microglia and contact duration of synapses with microglia. (c) Possible glial type‐specific synaptic tag, which is recognized by astrocytic MEGF10 or microglial TREM2

As described in the previous section, astrocytes would prefer smaller cell debris during phagocytosis (Damisah et al., 2020; Morizawa et al., 2017). However, because synapses are small structures, differences in synaptic size may not be a determinant. In contrast, the distance between glial cells and synapses could be a critical determinant. It is well known that astrocytes are located beside synapses as a component of the tripartite synapse to modulate synapse transmission (Figure 3a) (Araque et al., 1999). Therefore, astrocytes would have an advantage in synaptic phagocytosis when synapses are enwrapped by astrocytic processes. However, not all synapses are covered by astrocytes, and the coverage is dynamically altered during events such as development or parturition (Allen & Eroglu, 2017; Bernardinelli, Muller, & Nikonenko, 2014; Procko et al., 2011; Theodosis et al., 1986). More specifically, several studies have indicated that neuronal activity promotes synaptic coverage by astrocytes (Bernardinelli, Randall, et al., 2014; Genoud et al., 2006; Lushnikova et al., 2009). In addition to whether synapses are enwrapped by astrocytic processes, the frequency of synaptic surveillance by microglial processes and the contact duration of synapses with microglial processes may be important factors (Figure 3b). Microglial processes constantly extend to and touch synapses to survey the synaptic state (Wake et al., 2009). Microglial contact with synapses occurs in a neuronal activity‐dependent manner (Badimon et al., 2020; Wake et al., 2009). As with the *find‐me signal* released from dying or dead cells (Elliott et al., 2009; Koizumi et al., 2007), ATP is released from the synapses or perisynaptic astrocytes upon neuronal activation, resulting in the recruitment of microglial processes via the P2Y12 receptor (Badimon et al., 2020). Regarding a pathological condition, transient ischemia causes prolongation of microglial contact with synapses (Wake et al., 2009). After long contact by microglial processes, some synapses disappear, suggesting that recruitment of microglial processes to synapses may be critical for synaptic elimination. Collectively, neuronal activity‐ or state‐dependent spatiotemporal relationships between synapses and glial cells may determine whether the synapses are engulfed by astrocytes or microglia.

Nevertheless, a study reported that microglia were not able to compensate for reduced phagocytic activity of astrocytes in elimination of hippocampal excitatory synapses (J. H. Lee et al., 2021), suggesting that some synapses may have a fate to be phagocytosed specifically by astrocytes or microglia (Figure 3c). As discussed in the previous section, dead cells may originally have no preference, since PS exposure on the surface of dying or dead cells is the most initial tag for phagocytic clearance (D'Arcy, 2019; Fadok et al., 1992; Nagata, 2018; Westman et al., 2019). In contrast to dead cells, however, the molecular mechanisms of synaptic tagging for phagocytosis remain elusive. Although PS externalization and/or accumulation of complement molecules is thought to be an important cue (Gyorffy et al., 2018; Hong et al., 2016; T. Li et al., 2020; T. Li et al., 2021; J. Park et al., 2021; Schafer et al., 2012; Scott‐Hewitt et al., 2020), it is possible that other additional tag(s) may exist (Figure 1). MEGF10, which is expressed specifically by astrocytes, is a mammalian homolog of *Drosophila* Draper (Freeman et al., 2003). A group reported that Draper is a multi‐ligand receptor that recognizes both protein and lipoteichoic acid (Hashimoto et al., 2009; Kuraishi et al., 2009), the major constituent of gram‐positive bacterial walls, suggesting that astrocytic MEGF10 might target substances other than PS. Microglia‐specific phagocytic receptor TREM2 is a multi‐ligand receptor that recognizes a wide variety of ligands other than PS, such as other lipid species, nucleotides, Aβ oligomers, and proteins including apolipoprotein E (Atagi et al., 2015; Bailey et al., 2015; Kawabori et al., 2015; Y. Wang et al., 2015; Y. Zhao et al., 2018). Therefore, in addition to PS, unidentified tags, which are specifically recognized by astrocytic MEGF10 or microglial TREM2, might exist, providing a synapse with a selectivity to be engulfed by specific glial cells. Further studies on the tagging of unnecessary synapses may evaluate this possibility.

## 4 INTERACTIVE REGULATION BETWEEN ASTROCYTES AND MICROGLIA

**FIGURE 4:**
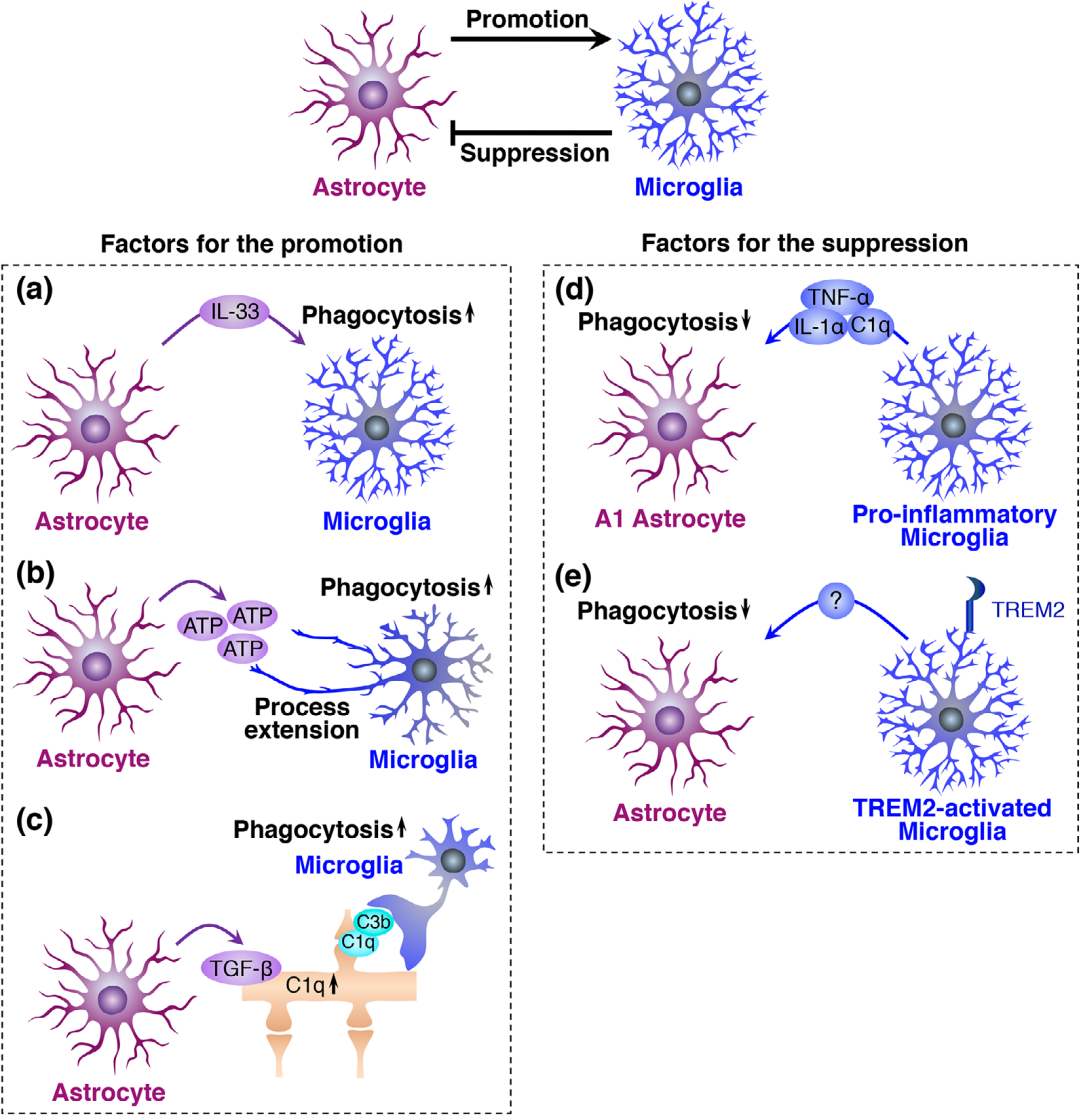
Interactive regulation between astrocytes and microglia, which promote microglial phagocytosis. Promotion of microglial phagocytosis by astrocytes (a–c) and suppression of astrocytic phagocytosis by microglia (d and e). (a) Astrocyte‐derived IL‐33 promotes microglial phagocytosis. (b) Astrocyte‐derived ATP promotes microglial phagocytosis. (c) Astrocyte‐derived TGF‐β1 stimulates neuronal C1q expression. Synaptic C3 deposition is enhanced, promoting microglial phagocytosis of synapses. (d) A specific combination of pro‐inflammatory molecules (IL‐1α, TNF‐α, and C1q) secreted from microglia suppress astrocytic phagocytosis. (e) TREM2 signal‐mediated activation of microglia suppresses astrocytic phagocytosis

Astrocytes and microglia can crosstalk in a soluble factor‐ or contact‐mediated manner, regulating each other's activity in both health and disease (Liddelow et al., 2020; Matejuk & Ransohoff, 2020; Vainchtein & Molofsky, 2020). Indeed, several studies have reported that astrocytes influence the phagocytic activity of microglia. During postnatal development, astrocytes around synapses secrete IL‐33 to enhance the microglial activity of synaptic pruning via its receptor IL1RL1 (Figure 4a) (Vainchtein et al., 2018). Microglia express purinergic receptors, such as P2X7, P2Y6, and P2Y12, whose downstream signal triggers microglial phagocytosis (Haynes et al., 2006; Koizumi et al., 2007; Rajbhandari et al., 2014). It is reported that activated astrocytes secreted ATP to enhance phagocytic activity of microglia via P2Y12 purinergic receptor (Figure 4b) (Xia et al., 2021). Astrocyte‐derived ATP also assists microglial phagocytosis by acting as a chemoattractant for microglia to the site of phagocytosis. After focal brain injury, astrocytes amplify extracellular ATP, which is initially leaked from damaged cells, to attract microglial processes (Figure 4b) (Davalos et al., 2005). Besides IL‐33 and ATP, astrocytes also secrete a variety of molecules that potentially promote microglial phagocytosis (Choi et al., 2014; Ponath et al., 2017; Verkhratsky et al., 2016). In addition to these direct effects on microglia, astrocytes indirectly support microglial phagocytosis during synaptic pruning (Figure 4c). In the developing retinogeniculate system, TGF‐β1 secreted from astrocytes stimulates C1q expression in retinal ganglion cells. Accordingly, C3 deposition on retinal ganglion cell synapses is promoted, resulting in enhanced synaptic pruning by microglia (Bialas & Stevens, 2013). Thus, astrocytes can directly or indirectly promote microglial phagocytosis.

Conversely, several reports have suggested that microglia inhibit astrocytic phagocytosis (Figure 4d and e). A specific combination of pro‐inflammatory molecules (IL‐1α, TNF‐α, and C1q) secreted from microglia induce neurotoxic “A1 astrocytes” (Liddelow et al., 2017), although this simplified classification of astrocytes is under debate (Escartin et al., 2021). A1 astrocytes exhibit suppressed phagocytic activity for synapses and myelin concomitantly with downregulation of phagocytic receptors such as MEGF10 and MERTK (Figure 4d) (Liddelow et al., 2017), indicating that microglia‐derived pro‐inflammatory molecules inhibit the phagocytic activity of astrocytes. Similar to this finding, TREM2 signal‐dependent microglial activation, which occurs during normal development, inhibits synaptic pruning by astrocytes (Figure 4e) (Jay et al., 2019). Astrocytes engulfed more synaptic elements in conventional *Trem2* knockout mice. The study further showed, in vitro that uptake of synaptosome by cultured astrocytes was inhibited when they were incubated for 24 hours with microglial conditioned media derived from wild‐type mice, not from the *Trem2* knockout mice, suggesting that TREM2 signal‐mediated activation of microglia stimulates secretion of some factor(s) to inhibit astrocytic phagocytosis. The inhibitory effects of microglia on astrocytic phagocytic activity have also been supposed when astrocytes engulf dead cells. As described in a previous section, intravital live imaging showed territories of astrocytes and microglia when cell death was induced in a single neuron. Both glial cells respect each other's phagocytic territory; however, astrocytes invade the primary microglial territory to engulf dead neurons upon microglial depletion (Damisah et al., 2020). Likewise, studies including ours revealed that astrocytes phagocytose dead cells in the absence or dysfunction of microglia (Abiega et al., 2016; Konishi et al., 2020; Punal et al., 2019), proposing the possibility that microglia may limit the phagocytic activity of astrocytes. Collectively, microglia may have a phagocytic advantage over astrocytes via inhibition of astrocytes (Figure 4d and e) as well as via activation by astrocytes (Figure 4a–c), which may be the reason microglial phagocytosis is more prominent overall than astrocytic phagocytosis.

## 5 CONCLUSIONS AND FUTURE PERSPECTIVES

Microglia have been regarded as phagocytes in the CNS from the period when Rio‐Hortega introduced the microglial concept (del Rio‐Hortega, 1932; Kettenmann et al., 2011). Although astrocytic phagocytosis was, to the best of our knowledge, first reported in the 1960s (Colonnier, 1964; McMahan, 1967; Mugnaini & Walberg, 1967), astrocytic phagocytosis was not highlighted until this decade. Astrocytes have phagocytic machinery even under physiological conditions (Cahoy et al., 2008; Konishi et al., 2020), and exert their phagocytic activity both in health and disease (Galloway et al., 2019; Jung & Chung, 2018). Although phagocytic targets reported to date are mostly common between astrocytes and microglia, astrocytes may have their own phagocytic targets, such as specific synapses (Figure 3c) (J. H. Lee et al., 2021). Regarding the size of phagocytic targets, astrocytes dislike large materials, in contrast to microglia (Damisah et al., 2020; Morizawa et al., 2017). Therefore, when we consider the phagocytic interplay between them, it would be worth noting the possible difference between astrocytes and microglia. However, knowledge of astrocytic phagocytosis, particularly their molecular mechanisms, remains limited compared to that of microglia. Also, it remains unclear whether or how astrocytic phagocytic activity is impacted by brain regions as well as intrinsic factors such as sex and age. Current techniques, such as high‐throughput single‐cell RNA‐seq (Armand et al., 2021), tri‐culture system of human pluripotent stem cell‐derived astrocyte/microglia/neuron (Guttikonda et al., 2021), live imaging of astrocytic phagocytosis using pH indicators (Byun & Chung, 2018), and molecular tracing of astrocytes‐microglia interaction (Clark et al., 2021), may help future studies on astrocytic phagocytosis.

Elucidation of glial phagocytosis may provide new therapeutic strategies. Regarding synapses, abnormal synaptic pruning by microglia during development causes an increased or decreased number of synapses, which is related to autism spectrum disorder and schizophrenia, respectively (Filipello et al., 2018; Sellgren et al., 2019; Zhan et al., 2014). In AD, aberrant synapse pruning by microglia is suggested to cause undesired synapse loss (Hong et al., 2016). Therefore, glial phagocytosis is highly related to psychiatric and neurodegenerative disease. However, the therapeutic approach in specific synapse elimination would be rather difficult (J. H. Lee et al., 2021; J. Park et al., 2021), and a simple enhancement of phagocytic activity may cause adverse effects (Hong et al., 2016; Paolicelli et al., 2017). In contrast, acceleration of dead cell clearance could be much easier and beneficial in an aged and diseased CNS. Microglial phagocytic activity declines along with aging and under some diseased conditions (Abiega et al., 2016; Baik et al., 2019; Galatro et al., 2017; Krabbe et al., 2013; Pluvinage et al., 2019), and promotion of astrocytic phagocytosis could be beneficial because astrocytes have the potential to compensate for the impaired microglial clearance of dead cells (Abiega et al., 2016; Damisah et al., 2020; Konishi et al., 2020; Punal et al., 2019).

To promote the clearance of dead cells, enhancement of lysosomal biogenesis as well as upregulation of phagocytic receptors in astrocytes is expected to be a therapeutic target (Chandra et al., 2018; Martini‐Stoica et al., 2018; Morizawa et al., 2017; Xiao et al., 2014). Although not a focus of this review, both astrocytes and microglia phagocytose Aβ (Itagaki et al., 1989; Koistinaho et al., 2004; Yamaguchi et al., 1998). In a mouse model of AD (APP/PS1 mouse), microglial phagocytic capacity declined according to the disease progression (Krabbe et al., 2013). Also, astrocytic uptake of Aβ is reduced according to the disease progression in 5XFAD mice (Iram, Trudler, et al., 2016), suggesting that astrocytes may not compensate for microglial impairment. However, studies demonstrated that enhancement of lysosomal biogenesis in astrocytes promotes clearance of Aβ and ameliorate amyloid plaque pathology in APP/PS1 or 5XFAD mice (Chandra et al., 2018; Xiao et al., 2014). Therefore, enhanced lysosomal biogenesis in astrocytes can compensate for impaired microglial clearance of Aβ in AD. These results also suggest the potential application of promotion of astrocytic lysosomal biogenesis for efficient clearance of dead cells.

Metabolic reprogramming of astrocytes could be another therapeutic target for accelerated clearance of dead cells. Regarding microglia, they have metabolic flexibility (Bernier et al., 2020), and the balance between glycolysis and mitochondrial oxidative phosphorylation significantly affects their phagocytic activity (Baik et al., 2019; Pan et al., 2019; Piers et al., 2020; Rubio‐Araiz et al., 2018). As described, microglial phagocytic capacity is considered to decline in AD. However, metabolic reprograming, which promotes glycolysis or a switch from glycolysis to oxidative phosphorylation, restores microglial phagocytic clearance of Aβ in APP/PS1 or 5XFAD mice (Baik et al., 2019; Pan et al., 2019). Although questions remain, such as whether and how energy metabolism regulates astrocytic phagocytic activity, it is highly expected that manipulations, which provide astrocytes with energy, promote astrocytic phagocytosis.

Further studies may provide clues for the therapeutic application of astrocytic phagocytosis to the efficient clearance of extracellular protein aggregates as well as dead cells in the CNS.

## AUTHOR CONTRIBUTIONS

**Hiroyuki Konishi:** Writing ‐ original draft preparation. **Schuichi Koizumi:** Writing ‐ review & editing. Hiroshi Kiyama: Writing ‐ review & editing.

## CONFLICT OF INTEREST

All authors declare no conflicts of interest.

## Data Availability

Data sharing is not applicable to this article as no new data were created or analyzed in this study.
